# Retraction Note: Fibulin-3 promotes osteosarcoma invasion and metastasis by inducing epithelial to mesenchymal transition and activating the Wnt/β-catenin signaling pathway

**DOI:** 10.1038/s41598-023-38918-9

**Published:** 2023-07-19

**Authors:** Songgang Wang, Dong Zhang, Shasha Han, Peng Gao, Changying Liu, Jianmin Li, Xin Pan

**Affiliations:** 1 Department of Orthopedics, Qilu Hospital, Shandong University, Jinan, 250012 China; 2 Department of Orthopedics, People’s Hospital of Zhangqiu, Zhangqiu, 250200 China; 3 Department of Orthopedics, People’s Hospital of Yinan, Linyi, 276000 China

Retraction of: *Scientific Reports* 10.1038/s41598-017-06353-2, published online 24 July 2017

The Editors have retracted this Article.

After publication of this Article it was brought to the Editors’ attention that some of the images appear to overlap with those in other published articles, where the data is partially attributed to different samples. Specifically:

- In Fig. 2C (right panel) and D (left panel) appear to partially overlap between the HOS-1 and HOS-29 groups;
- In Fig. 2D and 7A the image appears to be rotated;
- In Fig. 3C the “hFOB” group seems to overlap with Fig. 6D “non-infected” group;
- In Fig. 3E and 7B there appears to be a partial overlap between the “HOS” and “non-infected” (upper panel) groups;
- In Fig. 7A and B there appears to be a partial overlap between the “EFEMP1 shRNA” condition (top panel) and “HOS-29 neg control” (top panel) group;
- In Fig. 7B there appears to be a partial overlap between the “non-infected” (top panel) and “HOS-29 neg control” (top panel) groups;
- In Fig. 4E and 5A appear to partially overlap between the “HOS-1” conditions;
- In Fig. 6C the “HOS-1 neg control” group appears to partially overlap with Fig. 2C “HEC-1A” group in^1^;
- In Fig. 9A the “Slug” immunoblot appear to be partially overlapping with Fig. 10A “N-Cadherin” blot and 11A “E-Cadherin” blot in^1^;
- In Fig. 10B the “GSK3-beta pS9” blot seems to be overlapping with Fig. 4C “Cyclin-B1” blot in^2^.

The Editors reached out to the Authors to request raw data. The Authors provided the data which did not address the concerns. The Editors therefore no longer have confidence in the results presented in this Article.

Songgang Wang, Dong Zhang, Shasha Han, Peng Gao, Changying Liu, and Jianmin Li did not respond to the correspondence about this retraction. Xin Pan did not state explicitly whether they agree to or disagree with this retraction.
